# Dietary glycemic index and dietary glycemic load is associated with apelin gene expression in visceral and subcutaneous adipose tissues of adults

**DOI:** 10.1186/s12986-019-0389-9

**Published:** 2019-09-18

**Authors:** Emad Yuzbashian, Golaleh Asghari, Maryam Aghayan, Mehdi Hedayati, Maryam Zarkesh, Parvin Mirmiran, Alireza Khalaj

**Affiliations:** 1 Nutrition and Endocrine Research Center, Research Institute for Endocrine Sciences, Shahid Beheshti University of Medical Sciences, Tehran, Iran; 2 Department of Clinical Nutrition and Dietetics, Faculty of Nutrition Sciences and Food Technology, National Nutrition and Food Technology Research Institute, Shahid Beheshti University of Medical Sciences, P.O.Box: 19816-19573, Tehran, Iran; 3 Cellular and Molecular Endocrine Research Center, Research Institute for Endocrine Sciences, Shahid Beheshti University of Medical Sciences, P.O. Box: 19395-4763, Tehran, Iran; 4 Tehran Obesity Treatment Center, Department of Surgery, Shahed University, Tehran, Iran

**Keywords:** Glycemic index, Glycemic load, Carbohydrate, gene expression, Visceral and subcutaneous adipose tissue

## Abstract

**Background:**

Apelin, as an adipokine, plays an important role in the pathogenesis of insulin resistance and type 2 diabetes. This study aimed to determine whether the quality and quantity of dietary carbohydrates were associated with apelin gene expression in subcutaneous and visceral adipose tissues.

**Methods:**

In this cross-sectional study, 102 adults who underwent minor abdominal surgery were selected. Approximately 100 mg of subcutaneous and visceral adipose tissues were collected during the surgery to measure apelin gene expression. Anthropometric measurment, blood samples, and dietary intakes were collected before surgery. The dietary carbohydrate intake, glycemic index (GI), and glycemic load (GL) were determined.

**Results:**

The average apelin concentration was 269.6 ± 98.5(pg/mL), and 16.3% of participants were insulin resistant. There was a correlation between insulin (*p*-value = 0.043), Homeostatic Model Assessment for Insulin Resistance (HOMA-IR)(*p*-value = 0.045) and apelin gene expression in visceral adipose tissue. There was a positive association of apelin gene expression with dietary GI and GL after adjustment for age, sex, and waist circumference in visceral and subcutaneous adipose tissues(*p* < 0.05). Apelin gene expression in visceral(*p* = 0.002) and subcutaneous(*p* = 0.003) adipose tissues was directly associated with foods with a higher GI. There was no association between total carbohydrate intake and apelin gene expression in both visceral and subcutaneous adipose tissues.

**Conclusions:**

Dietary GI and GL, not total carbohydrate intake, were positively associated with apelin gene expression in both visceral and subcutaneous adipose tissues. Future studies are warranted to illustrate the chronic and acute effect of carbohydrate quality on apelin homeostasis.

**Electronic supplementary material:**

The online version of this article (10.1186/s12986-019-0389-9) contains supplementary material, which is available to authorized users.

## Background

More attention has been paid to the role of adipocytes in the body homeostasis in the recent decade. Adipose tissue acts as an endocrine organ which has an important effect on insulin sensitivity and regulates energy homeostasis through adipocyte-derived regulatory hormones termed adipokines [1, 2]. Apelin, as an adipokine, is a 36 amino-acid bioactive peptide that in humans is encoded by the apelin gene and located on chromosome Xq25–26.1. It is a key regulator in glucose and lipid metabolism [3] and plays an important role in the pathogenesis of insulin resistance and type 2 diabetes [4, 5]. Apelin regulates glucose homeostasis by increasing glucose uptake, enhancing insulin sensitivity in adipocytes [3] and inhibiting adipocyte lipolysis [6, 7].

Carbohydrates are the primary source of energy in the human diet. Glycemic index (GI) and glycemic load (GL), as measurements of carbohydrate quality, provide an additional benefit compared to carbohydrate counting alone [8]. Dietary glycemic measures may affect body homeostasis [9] and contribute to the adipose-related pathways [10].

In our previous systematic review, we concluded that diet could alter apelin gene expression and concentration by mediating on plasma insulin levels [11]. It is well-established that insulin concentration is the important stimulator of apelin gene expression in adipose tissue [12]. Thus, we hypothesized that habitual dietary intake of carbohydrates, including quality and quantity, might have a distinguishing role in the prediction of apelin gene expression in adipose tissue by chronic manipulating of insulin concentration.

According to recent studies, apelin concentration may be affected by dietary intake in both human and animals [13, 14]. However, these studies mostly focused on dietary fatty acid, and data regarding dietary carbohydrate was rare. Thus, this study aimed to determine whether quality and quantity of dietary carbohydrates were associated with apelin gene expression of in subcutaneous and visceral adipose tissues.

## Materials and methods

### Participants

In this cross-sectional study, 102 participants aged more than 20-years, who had been admitted for elective abdominal surgery including appendectomy and hernia repair, were selected from Mostafa Khomeini and Khatam Al-Anbia Hospitals, Tehran, Iran. All participants were hospitalized for less than three days. Individuals were eligible for inclusion if they had no known medical illnesses such as diabetes mellitus or cancer, did not take lipid-lowering or anti-obesity medications, were not pregnant or lactating woman, and not on special diets. Approximately 100 mg of subcutaneous and visceral adipose tissues were collected during the surgery. All information and questionnaires were completed before surgery.

The ethics committee of the Research Institute for Endocrine Sciences (RIES) of Shahid Beheshti University of Medical Sciences approved the study (NO: IR.SBMU.ENDOCRINE.REC.1395.372), and the study was conducted in accordance with the Declaration of Helsinki, as well as our institutional guidelines. Written informed consent was obtained from all participants.

### Dietary measurement

Using a valid and reliable semi-quantitative food frequency questionnaire (FFQ), expert interviewers gathered regular dietary intakes of each participant during the past year, on a daily, weekly, or monthly basis, and according to household measures. Then, portion sizes of consumed foods were converted to grams. Energy and nutrient contents were obtained from the US Department of Agriculture (USDA) food composition tables (FCT) because the Iranian FCTs were incomplete; although, the Iranian FCTs were used for traditional food items (like Kashk). The reliability and validity of the FFQ was evaluated in a previous study and indicated that the FFQ provides reasonably valid measures of the long-term average [15].

In the current study, we considered total carbohydrate intake and dietary GI and GL. Then, the food types were categorized based on their GI and GL into three different groups including high (≥70), medium (56–69) and low (≤55) GI foods (Additional file 1: Table S1) [16].

Dietary GI and GL were derived from the FFQ as follow [16]:

$$ \mathbf{Dietary}\ \mathbf{GI}=\left[\left(\mathrm{carbohydrate}\ \mathrm{content}\ \mathrm{of}\ \mathrm{each}\ \mathrm{food}\ \mathrm{item}\right)\times \left(\mathrm{number}\ \mathrm{of}\ \mathrm{servings}/\mathrm{d}\right)\times \left(\mathrm{GI}\right)\right]/\mathrm{total}\ \mathrm{daily}\ \mathrm{carbohydrate}\ \mathrm{intake} $$

$$ \mathbf{Dietary}\ \mathbf{GL}=\left(\mathrm{carbohydrate}\ \mathrm{content}\ \mathrm{of}\ \mathrm{each}\ \mathrm{food}\ \mathrm{item}\right)\times \left(\mathrm{number}\ \mathrm{of}\ \mathrm{servings}/\mathrm{d}\right)\times \left(\mathrm{GI}\right) $$

### Quantitative real-time polymerase chain reaction analysis of gene expression

We extracted total RNA from visceral and subcutaneous fat tissues according to the manufacturer’s protocol by using RNX-plus solution (Cinnagen, Iran). The quality of the extracted RNA was assessed by NanoDrop spectrophotometer (Thermo Fisher Scientific, Waltham, USA) and the ratio of absorption (260/280 nm) of all preparations were in an acceptable range.

In order to remove traces of genomic DNA before complementary DNA (cDNA) synthesis, total RNA was treated with DNase I. The cDNA synthesis kit (Thermo Scientific, USA) was used according to the manufacturer’s recommendations. The product was stored at − 20 °C for further investigations.

Primers based on the sequences of the National Center for Biotechnology Information (NCBI) GenBank database were checked by Genrunner Software (version 3.05). The glycerinaldehyde-3-phosphate dehydrogenase GAPDH gene was used as the reference gene for normalization across samples. Primer sequences of apelin were as follows:

- apelin Forward: 5′- TCT GAC CCC CAA AGA TGA TG-3′;
- apelin Reverse: 5′- CTC GGA GAA TTA GTT TAG GAT ATT TCA-3′;

The Real-Time quantitative PCR (qPCR), carried out using a Real-Time PCR instrument (Rotor-Gene 6000, Sydney, Australia), was performed in 25 μL volumes containing 12.5 μL 2X SYBR Green Master mix (Thermo Scientific, USA), 0.3 μL forward primers, 0.3 μL reverse primers, 8.9 μL RNase- free water, and 3 μL of the cDNA. For each gene, samples were run in duplicate for inter assay control along with GAPDH (housekeeping) and the non-template control (NTC); qPCR amplification was performed with the following thermal cycling conditions: 5 min at 95 °C for denaturation, followed by 45 cycles at 95 °C for 30 s, 60 °C for 30 s and 72 °C for 30 s for annealing, amplification, and quantification. The relative expression of apelin in each sample was calculated based on its threshold cycle (Ct), normalized to the Ct of the reference gene. All qPCR laboratory procedures were performed according to the MIQE guidelines [17].

### Anthropometric and laboratory measurements

Weight was measured in minimum clothing to the precision of 0.1 kg on a SECA digital weighing scale (Seca 707; Seca Corporation, Hanover, Maryland; range 0.1–200 kg), and height was measured to the nearest 0.1 cm while barefoot. Body mass index (BMI) was calculated as weight (kg) divided by square of height (m^2^). Waist circumference (WC) was measured to the nearest 0.5 cm, using a measuring tape while in standing position and after a gentle respiration, at the level of the umbilicus.

Physical activity was assessed during interviews, using the long forms of the reliable and validated Persian version of the International Physical Activity Questionnaire (IPAQ) [18]. The concept of metabolic equivalents (MET) was used to measure energy expenditure. Low level of physical activity was considered as equivalent metabolic task < 600 min/wk.

Arterial blood pressure (BP) was measured for each participant using a mercury sphygmomanometer. Systolic blood pressure (SBP) was determined by the onset of the tapping Korotkoff sound and diastolic blood pressure (DBP) as the disappearance of this sound. Blood pressure was measured twice, and the average was considered as the participant’s BP.

Blood samples were drawn from all study participants between 7:00 and 9:00 AM after 12–14 h of overnight fasting. All the blood analyses were done at the TLGS research laboratory on the day of blood collection. Fasting plasma glucose (FPG) was measured by the enzymatic colorimetric method using glucose oxidase. Inter- and intra-assay coefficients of variation (CV) were both 1.0% for FPG. Serum triglycerides (TGs) were assayed using an enzymatic colorimetric method with glycerol phosphate oxidase. Inter- and intra-assay CV for TGs were 0.4 and 2.1%, respectively. These analyses were performed using commercial kits (Pars Azmoon, Tehran, Iran) and a Selectra 2 autoanalyzer (Vital Scientific, Spankeren, The Netherlands). Insulin was measured using the enzyme-linked immunosorbent assay (ELISA) with Mercodia kits (Uppsala, Sweden). Inter- and intra-assay CVs of insulin were 1.7 and 2.3, respectively.

### Statistical analysis

Normality of variables was tested by the histogram and Kolmogorov-Smirnov test. Because plasma TGs and insulin were skewed, log transformation was used. Quantitative variables were described as mean ± standard deviation (SD) or median with interquartile range, and qualitative variables were reported as percentage. Relative mRNA expression levels were determined for each sample separately using internal reference genes GAPDH with the formula 2^-(ΔCt)^, where ΔCt is Ct (target gene) − mean of Ct (reference genes) [19] . In order to adjust energy, we used the residual method for all dietary exposures including total carbohydrate, GI, GL, and food groups. Then, these variables were used for all statistical analysis [20].

The association of apelin gene expression according to tertiles of energy-adjusted dietary GI in visceral and subcutaneous adipose tissues was determined using LSD. The correlation of apelin gene expression in visceral and subcutaneous adipose tissues with energy-adjusted dietary carbohydrate was performed by bivariate correlation. To investigate the trend of variables according to tertiles of GI, the linear regression and chi-square test was used for continuous and categorical variables, respectively. Adjusted linear regression tests were performed in two models (model 1 adjusted for age and sex, and model 2 additionally adjusted for WC) to evaluate possible associations of dietary carbohydrates, GI, GL, and high, medium and low glycemic foods with apelin gene expression. Both unstandardized (95% confidence interval) and standardized β (STZ β) were reported. Before running multivariable linear models, the interaction terms between age, sex and WC, and dietary exposures were examined. No interaction was observed between considered covariates and dietary carbohydrates, GI, GL, and high, medium and low glycemic foods. All data were analyzed using the Statistical Package for Social Sciences program (SPSS) (version 15.0; SPSS Inc., Chicago IL) and *P*-values < 0.05 were considered statistically significant.

## Results

The current study consisted of 102 non-diabetic participants characterized by a mean age of 41.7 ± 14.6 years, and mean BMI of 35.2 ± 10.7 kg/m^2^. The average apelin concentration was 269.6 ± 98.5 (pg/mL). Among participants, 16.3% presented insulin resistance.

The biochemical, anthropometric, and dietary characteristics of participants according to tertiles of dietary GI are summarized in Table 1. Insulin concentration, HOMA-IR, and prevalence of insulin resistance were significantly increased in subjects across the tertiles of dietary GI.

**Table 1:** Demographic, anthropometric, dietary intake and serum biochemical parameters according to the tertiles of dietary glycemic index^a^

	Dietary glycemic index tertiles			
	T1	T2	T3	*P* value^b^
Age (years)	43.3 ± 14.4	43.4 ± 14.2	38.3 ± 14.2	0.252
Female (%)	85.3	70.6	78.8	0.514
Low physical activity (%)	47.1	44.1	42.4	0.882
Body mass index (kg/m^2^)	33.8 ± 10.7	36.6 ± 10.8	35.3 ± 10.6	0.561
Waist Circumference	107.1 ± 22.5	113.2 ± 22.0	107.6 ± 24.3	0.491
Fasting plasma glucose (mg/dl)	86.7 ± 12.1	86.7 ± 10.9	87.6 ± 8.3	0.925
Insulin (μU/mL)	6.0 (4.4–9.8)	5.8 (3.1–8.1)	9.4 (5.6–19.6)	0.007
HOMA-IR	1.74 ± 1.5	1.47 ± 1.1	3.3 ± 4.3	0.012
Insulin resistant (%)	11.8	5.9	22.3	0.020
Triglycerides (mg/dl)	68.0 (61.7–90.2)	66.5 (60.2–83.7)	72.5(65.0–87.5)	0.463
Apelin (pg/mL)	263.2 ± 68.9	244.4 ± 82.6	302.6 ± 120.6	0.048
Total energy intake (kcal)	2778 ± 628	2951 ± 849	2896 ± 1027	0.694
Total carbohydrate (% of energy)	54.6 ± 6.1	57.8 ± 8.2	57.7 ± 6.7	0.121
Total carbohydrate (g/d)	397.9 ± 53.1	403.4 ± 53.2	421.4 ± 54.9	0.181
Dietary glycemic index	48.1 ± 5.5	56.5 ± 1.7	65.7 ± 6.8	< 0.001
Dietary glycemic load	188.8 ± 33.4	232.3 ± 40.8	275.8 ± 47.5	< 0.001
High glycemic foods (serv/d)	6.9 ± 2.2	8.8 ± 3.6	11.7 ± 4.1	< 0.001
Medium glycemic foods (serv/d)	1.3 ± 0.6	0.9 ± 1.9	1.1 ± 1.5	0.258
Low glycemic foods (serv/d)	11.3 ± 4.6	11.3 ± 6.0	8.4 ± 11.0	0.006
Protein (% of energy)	14.7 ± 2.8	14.2 ± 2.5	13.7 ± 2.7	0.328
Fat (% of energy)	33.6 ± 5.9	30.8 ± 6.7	30.7 ± 5.1	0.090

^a^Data are represented as mean ± standard deviation or median (interquartile range) for continuous variables and percent for categorically distributed variables

^b^linear regression was used for continuous variables and chi-square test for categorical variables

The residual model was used to adjust total energy intake for dietary exposures

**Fig. 1:**
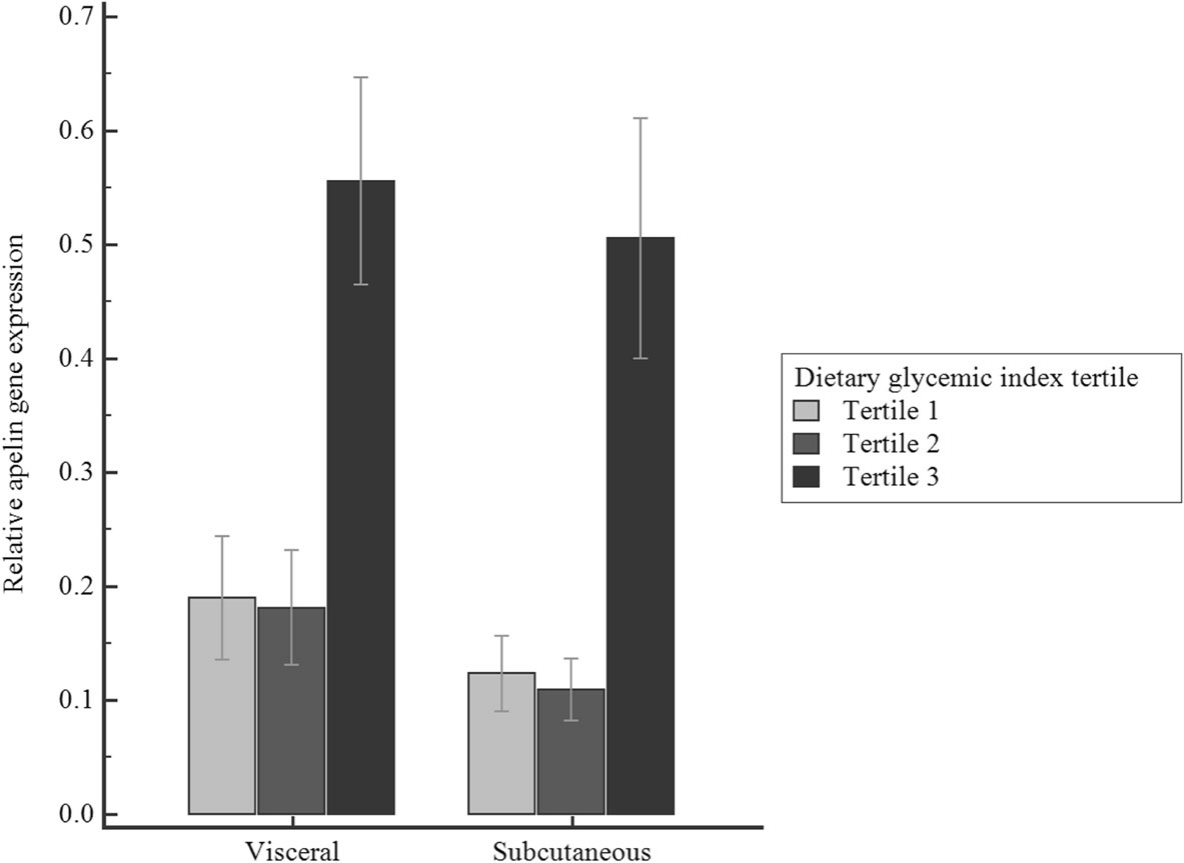
Distribution of relative apelin gene expression in visceral and subcutaneous adipose tissues according to the tertile of energy-adjusted dietary glycemic index (GI). Results are expressed as mean ± SEM. *P*-values for the association of apelin gene expression in the lowest tertile in comparison with medium and highest tertile of energy adjusted dietary GI in visceral adipose tissue were 0.928 and < 0.001, respectively. *p*-values for the association of apelin gene expression in the lowest tertile in comparison with the medium and highest tertile of energy-adjusted dietary GI in subcutaneous adipose tissue were 0.878 and < 0.001, respectively

Participants in the highest tertile of dietary GI had more apelin gene expression in visceral and subcutaneous adipose tissue (Fig. 1). No significant differences were observed in mean BMI across dietary GI groups. There was a significant correlation of insulin (*r* = 0.201, *p*-value = 0.043) and HOMA-IR (*r* = 0.210, *P* value = 0.045) with apelin gene expression only in visceral adipose tissue (data not shown).

**Fig. 2:**
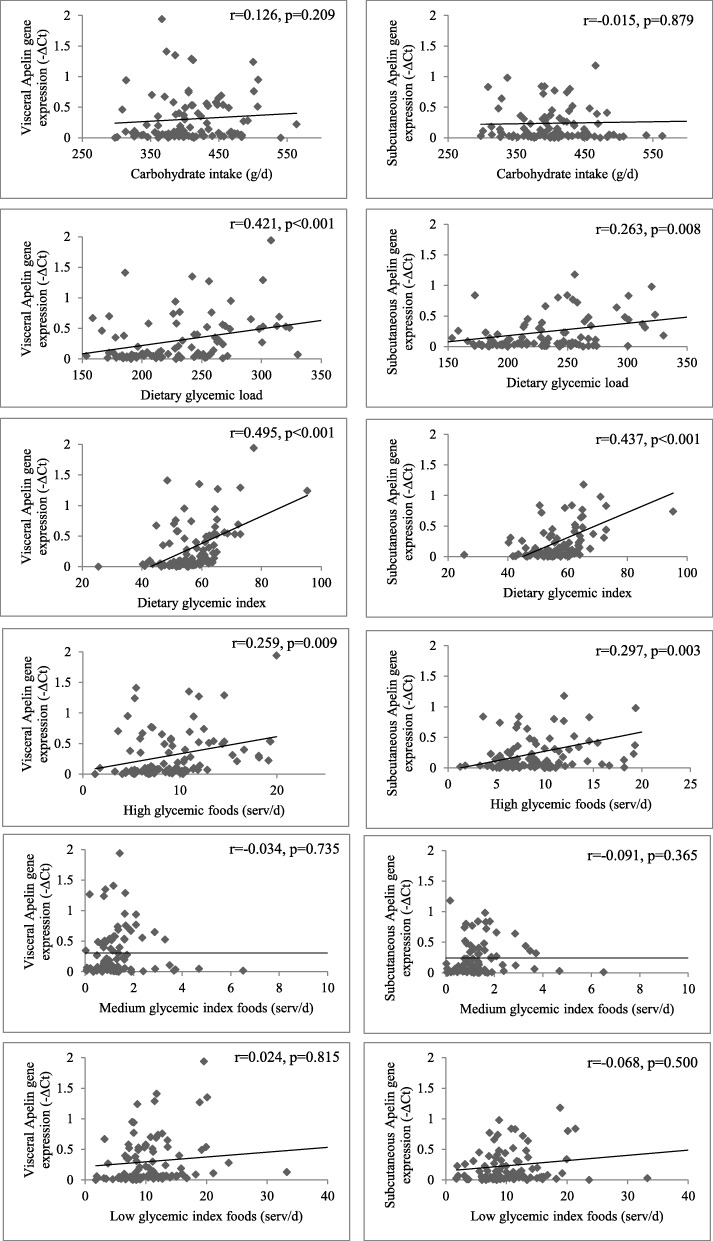
Correlation between apelin gene expression in visceral and subcutaneous adipose tissues and energy-adjusted dietary carbohydrate intake throughout the study participants

Energy-adjusted dietary GI was positively correlated with apelin gene expression in visceral (*r* = 0.495, *P* < 0.001) and subcutaneous (*r* = 0.437, *P* < 0.001) adipose tissues. Furthermore, dietary GL had a significant direct correlation with both visceral (*r* = 0.421, *P* < 0.001) and subcutaneous (*r* = 0.262, *P* = 0.0.008) apelin gene expression. A positive correlation was also observed between intake of high GI foods and apelin gene expression in both visceral and subcutaneous adipose tissues (Fig. 2).

The association between dietary GI and apelin gene expression remained significant upon adjustment for age, sex and WC in the visceral (STZ β = 0.402, *P* < 0.001) and subcutaneous (STZ β = 0.477, *P* < 0.001) adipose tissues. Furthermore, there was a significant positive association between dietary GL and apelin gene expression in both visceral (STZ β = 0.224, *P* = 0.005) and subcutaneous (STZ β = 0.350, *P* = 0.029) adipose tissue. After adjusting for confounders, each standard deviation higher intake of foods with higher GI, was directly associated with apelin gene expression in visceral (STZ β = 0.253, *P* = 0.002) and subcutaneous (STZ β = 0.255, *P* = 0.003) adipose tissue (Table 2). Foods which were mainly contributed to high GI category including date and Lavash were directly associated with apelin gene expression in both adipose tissues (Additional file 1: Table S2).

**Table 2:** Regression coefficient of total carbohydrate, dietary glycemic index, and dietary glycemic load with apelin gene expression in adipose tissues

	Subcutaneous			Visceral		
	Β (95% CI)	STZ β	*P* value	Β (95% CI)	STZ β	*P* value
Total carbohydrate (per 54.1 g/d)						
Age and sex adjusted	0.000 (−0.002 to 0.002)	− 0.006	0.952	0.001 (− 0.001 to 0.002)	0.081	0.426
Age, sex, and WC Adjusted	0.000 (− 0.002 to 0.002)	− 0.022	0.839	0.000 (− 0.002 to 0.002)	−0.018	0.865
The dietary glycemic index (per 8.8)						
Age and sex adjusted	0.020 (0.011 to 0.029)	0.419	< 0.001	0.023 (0.015 to 0.035	0.481	< 0.001
Age, sex, and WC Adjusted	0.024 (0.013 to 0.034)	0.428	< 0.001	0.024 (0.013 to 0.034)	0.437	< 0.001
Dietary glycemic load (per 53.9)						
Age and sex adjusted	0.002 (0.000 to 0.003)	0.257	0.010	0.003 (0.001 to 0.004)	0.349	< 0.001
Age, sex, and WC Adjusted	0.002 (0.000 to 0.004)	0.246	0.029	0.003 (0.001 to 0.004)	0.395	0.005
High glycemic foods (per 3.9 serv/d)						
Age and sex adjusted	0.030 (0.010 to 0.051)	0.284	0.004	0.028 (0.007 to 0.049)	0.261	0.009
Age, sex, and WC Adjusted	0.034 (0.012 to 0.056)	0.306	0.003	0.036 (0.014 to 0.049)	0.328	0.002
Medium glycemic foods (per 1.5 serv/d)						
Age and sex adjusted	−0.012 (−0.070 to 0.047)	−0.041	0.692	−0.015 (−0.075 to 0.044)	−0.053	0.607
Age, sex, and WC Adjusted	−0.018 (− 0.083 to 0.047)	−0.060	0.578	−0.018 (− 0.082 to 0.047)	−0.059	0.588
Low glycemic foods (per 7.6 serv/d)						
Age and sex adjusted	−0.004 (− 0.015 to 0.007)	−0.076	0.455	0.000 (−0.011 to 0.012)	0.009	0.933
Age, sex, and WC Adjusted	−0.004 (− 0.011 to 0.005)	−0.142	0.180	0.002 (−0.017 to 0.011)	0.072	0.492

STZ β; standardized β, WC; waist circumference

The residual model was used to adjust total energy intake for dietary exposures

## Discussion

In the present study, dietary GI and dietary GL were directly associated with the apelin gene expression in both visceral and subcutaneous adipose tissue after controlling for age, sex, and WC. Moreover, habitual intake of high GI food had a positive association with subcutaneous and visceral apelin gene expression; per 3.9 serv/d increase in intake of high GI foods, apelin gene expression in subcutaneous and visceral adipose tissues increased 0.253 and 0.255 unit, respectively.

To the best of our knowledge, this was the first study which focused on the association of dietary quality and quantity of carbohydrate with apelin gene expression in human fat depots. However, a number of studies have investigated the relationship between different feeding patterns and apelin concentration and gene expression in both human and animals [13, 21–23]. In interventional studies, a caloric-restricted diet could decrease both the apelin concentration and gene expression in human [13, 21]. Castan laurel et al., have found that 12 weeks hypocaloric weight-reducing diet in 20 obese women was associated with increased plasma and adipose tissue expression of apelin gene [21]. Nevertheless, Celik et al., found that Ramadan fasting did not affect the apelin concentration in healthy men [22]. Moreover, apelin gene expression increased in the subcutaneous adipose tissue in response to a high-fat diet in rats [23]. It seems that the results of these studies are inconsistent and most of them had focused on the restricted calorie intakes and dietary fatty acids, and none of them had considered the dietary carbohydrate content as a probable factor which affects apelin concentration and gene expression.

Carbohydrates are the primary nutrient contributing to the supply of energy requirements; constituting 45–65% of the energy intake in a regular diet. It should be noted that dietary GI and GL imply some other aspects of dietary quality, which can partly reflect dietary characteristics. It is possible that higher carbohydrate intake may be substituted with dietary fat or protein while maintaining a constant energy intake [24]. Therefore, dietary composition might be a factor which can influence adiposities. An interesting point of our study was that carbohydrate quality, not total carbohydrate intake, was significantly associated with apelin gene expression, and emphasizing the importance of food quality instead of quantity.

The GI and GL represent different aspects of this macronutrient and are used to characterize the capability of food increasing serum glucose concentrations [25]. Foods with a relatively high GI can potentially affect body composition [26], and impact on specific metabolic processes, (such as lipolysis and lipogenesis) by increasing insulin to a greater extent or for a longer time [27, 28]. In the current study, we observed that high dietary GI foods had a positive relationship with apelin gene expression in both adipose tissues. Physiological alteration in response to low GI against high GI dietary intervention indicated that white adipose tissue gene expression was altered in pathways involving insulin sensitivity and fatty acid metabolism [28]. Furthermore, it was revealed that peroxisome proliferator-activated receptor-gamma (PPAR-γ), an important transcription factor in the regulation of apelin [29], showed an increase with high GI diet [28].

Although none of the participants in the current study had diabetes, by increasing the tertiles of dietary GI, insulin resistance significantly increased in the study population. Evidence showed that insulin resistance induced by a high-fat diet caused an increase in both apelin concentration and gene expression in adipose tissues [14, 30]. Moreover, apelin concentration and gene expression in mice has been reported to be regulated according to the severity of insulin resistance, suggesting a link between apelin and glucose homeostasis [31]. Dray et al. have shown that exogenous glucose promotes the luminal secretion of apelin, when administered to mice by gavage [32]. Regular consumption of meals with high GI increased 24-h blood glucose and insulin levels [33]. Since apelin concentration might be regulated by insulin concentration, dietary factors which influence glucose homeostasis can up-regulate apelin gene expression.

Our study has some limitations. Because of the cross-sectional design of the study, we could not assess the causality of the study. However, as it is less likely that apelin gene expression in fat depots influences the quality and quantity of carbohydrate intakes, we consider our inference that carbohydrate intakes may have primary effects on apelin gene expression plausible. Secondly, since there was no complete Iranian FCT, we had to use the USDA FCT. Third, another limitation of this study was the non-random selection of participants. Therefore, the results may not be representative of the population. The nature of the non-random sample in our study may also lead to a selection bias which warrants caution in interpreting the results. Presence of convenience sampling can prevent generalization of the findings to the broader population and become less representative of the intended population as time goes on. In order to be comparable and generalizable, the findings in future studies must consider recruitment of samples. Fourth, despite controlling critical potential confounders, several other confounders may still affect the association between dietary carbohydrate intake and apelin gene expression. This study also has its strength; it was the first study to provide data on dietary GI and its association with apelin gene expression. Also, the observational design of the current study reflected long-term habitual high GI food on apelin gene expression.

## Conclusion

In conclusion, dietary GI and GL, not total carbohydrate intake, was associated with apelin gene expression in both visceral and subcutaneous adipose tissues. Future studies are warranted to illustrate the chronic and accurate effect of carbohydrate quality on apelin homeostasis.

## Additional file

Additional file 1: **Table S1**. Foods contributed to making up different glycemic categories. **Table S2**. Foods substantially contributed to each GI category and apelin gene expression in adipose tissues. (DOCX 17 kb)

## Data Availability

All data generated or analyzed during this study are included in this published article.

## Abbreviations

BMI: body mass index
BP: blood pressure
CV: coefficients of variation
DBP: diastolic blood pressure
ELISA: enzyme-linked immunosorbent assay
FCT: food composition tables
FFQ: food frequency questionnaire
FPG: Fasting plasma glucose
GI: glycemic index
GL: glycemic load
IPAC: International Physical Activity Questionnaire
MET: metabolic equivalents task
SBP: Systolic blood pressure
SD: standard deviation
SPSS: Statistical Package for the Social Sciences program
STZ β: standardized β
TGs: triglycerides
USDA: US Department of Agriculture
WC: waist circumference
