# Supplementary material for: Dietary glycemic index and dietary glycemic load is associated with apelin gene expression in visceral and subcutaneous adipose tissues of adults
Source: Nutr Metab (Lond). 2019 Sep 18;16:68. doi: 10.1186/s12986-019-0389-9 (PMC6751847; doi:10.1186/s12986-019-0389-9)
Supplement: Supplementary file 1 — Table S1. Foods contributed to making up different glycemic categories. Table S2. Foods substantially contributed to each GI category and apelin gene expression in adipose tissues. (DOCX 17 kb) [file 12986_2019_389_MOESM1_ESM.docx]

| Additional file 1: Table S1. Foods contributed to making up different glycemic categories | | |
| --- | --- | --- |
| Low glycemic food | Medium glycemic food | High glycemic food |
| Barbari (whole and dark breads)  Sangak(whole and dark breads)  Taftoon(whole and dark breads)  Cooked angel hair pasta  Reshteh  lentil  bean  chickpea  soy bean  Mung bean  split peas  pizza  low fat milk  whole fat milk  cocoa milk  plain yogurt  whole yogurt  cheese  Doogh (Yogurt drink)  Kashk  Pear  Cherry  Apple  Grape fruit  Prune (yellow and red)  fig, mulberry, peach and apricot | Cooked pasta  Cooked barely and bulgur  Broad bean  Cooked green pea  Cooked carrot  Peach  Nectarine  Grapes  Orange  Persimmon  Tangerine | Lavash (white bread)  Baguette  Cooked rice  Potato  French fries  Biscuits  Cracker  Cake  Popcorn  Corn  Ice cream (plain and traditional (high fat))  Pumpkin  Cantaloupe  Melon  Watermelon  apricot,  fig  kiwi  dates  banana  orange juice  raisin  berry  cube sugar, Noghl  sugar  honey  jam  cola  All soft and sweet drinks,  pastries (non-crème  and creamy)  candy |

| Additional file 1: Table S2. Foods substantially contributed to each GI category and apelin gene expression in adipose tissues | | | | | | |
| --- | --- | --- | --- | --- | --- | --- |
|  | Subcutaneous | | | Visceral | | |
|  | Β (95%CI) | STZ β | P value | Β (95%CI) | STZ β | P value |
| **High glycemic foods category** |  |  |  |  |  |  |
| Rice | 0.016 (0.008 to 0.041) | 0.136 | 0.193 | 0.015 (0.010 to 0.042) | 0.123 | 0.251 |
| Lavash (White bread) | 0.035 (0.005 to 0.075) | 0.187 | 0.083 | 0.045 (0.006 to 0.084) | 0.245 | 0.023 |
| Date | 0.247 (0.082 to 0.412) | 0.303 | 0.004 | 0.258 (0.096 to 0.420) | 0.324 | 0.002 |
| **Medium glycemic foods category** |  |  |  |  |  |  |
| Pasta | -0.089 (-0.402 to 0.223) | -0.060 | 0.570 | -0.066 (-0.374 to 0.242) | -0.045 | 0.890 |
| Grapes | -0.016 (-0.268 to 0.235) | -0.013 | 0.899 | -0.052 (-0.300 to 0.195) | -0.045 | 0.675 |
| Peach | -0.058(-0.253 to 0.136) | -0.063 | 0.553 | -0.059 (-0.251 to 0.133) | -0.065 | 0.542 |
| **Low glycemic foods category** |  |  |  |  |  |  |
| Sangak (whole bread) | 0.032 (-0.013 to 0.078) | 0.146 | 0.164 | 0.034 (-0.011 to 0.079) | 0.157 | 0.137 |
| Taftoon (whole bread) | 0.031 (-0.003 to 0.066) | 0.188 | 0.074 | 0.007 (-0.028 to 0.041) | 0.042 | 0.693 |
| Yogurt (low fat) | 0.147 (-0.548 to 0.843) | 0.046 | 0.675 | 0.298 (-0.385 to 0.982) | 0.096 | 0.388 |

Adjusted for age, sex, and waist circumference
